# Notes on Obstetrics and Gynæcology

**Published:** 1875-09

**Authors:** E. N. Brush


ART. II.—*Notes on Obstetrics and Gynæcology.* By E. N. BRUSH, M. D.

1. On the Relative Frequency of the Different Cranial Positions. By JOSEPH GRIFFITH SWAYNE, M. D. (*Obstetrical Journal of Great Britain and Ireland,* Sept, 1875.)

2. On the Means of Ascertaining the Length of Gestation, by Measuring the Fœtus and Gravid Uterus during the Second Period of Pregnancy. By VASSILY SUTUGIN, M. D., St. Petersburg. (*Trans. Obstet. Society, Edinburgh.*)

3. The Diagnosis of Ovarian Cysts and the Indications for their Treatment. By DR. RHEINSTAEDTER, Cologne. (*Berliner Klinischer Wochenschrift,* May 31, 1875.

I. Dr. Swayne's paper is based on the observation of one thousand cases of cranial presentation. These observations were carefully made with a view of testing the accuracy of certain conclusions advanced in a paper read in 1852, on the "Varieties of Cranial Presentation." The conclusions made in 1852 were drawn from a record of 286 cranial presentations, of which 247 were of the first position, viz.: with the occiput towards the left acetabulum; 28 of the second, with the occiput towards the right acetabulum; 3 of the third, with the forehead toward the left acetabulum; and 8 of the fourth, with the forehead toward the right acetabulum. These figures conflicted with the generally accepted views of Britis accoucheurs that the third was, next to the first, the most common position, and that the second was the most rare. This was the teaching of Naegele. Baudelocque and most French obstetricians held a different view, but Naegeli accounted for the discrepancy by supposing that cases which had been put down as of the second position had originally been of the third, but had altered during labor. The statistics of Swayne in 1852, and the researches of West, and Leishman, whose work on the "Mechanism of Parturition" is well known, led to a reconsideration of the teachings of Naegele, and a doubt as to their correctness arose in many minds.

The record of the 1,000 cases since observed by Dr. Swayne shows the following proportion:

| | |
|---|---|
| First position | 792 |
| Second " | 152 |
| Third " | 19 |
| Fourth " | 37 |
| Total | 1,000 |

This record bears out the deductions made from the previous cases as to the frequency of the different positions.

In reference to the change said to occur from the third to the second position the author says: In the 19 cases of the third position the presentation remained unaltered in 8. The presentation altered in 5 spontaneously, changing to the second. In the remaining six the position was changed by manipulation to the second.

The deductions made from these cases are the same as those arrived at in 1852, viz.: "that the second position is next in order of frequency to the first, and that the fourth occurs more often than the third." The author is also of the opinion that in the third and fourth positions the occipito-posterior position is more apt to remain throughout the labor than to change spontaneously.

II. It is impossible to make any abstract of this paper which would embrace all the points set forth in the author's scheme of measurement. Following in the lead of Hecker Schroeder and Ahfield he has endeavored, by a series of observations upon the period of gestation as determined by the last catamenia, the corresponding size of the womb and the general development of the fœtus, to arrive at some approximate standard by which the length of gestation may be determined. In the course of the paper ten tables are given, the results of various measurements and weights made during the course of pregnancy and after delivery. Without entering into the details of these, we give the conclusions reached, hoping that our readers may have the opportunity of seeing the article entire. These deductions are:

1. The employment of the pelvimeter for measuring the height of the gravid uterus gives more trustworthy results than the use of the tape measure.

2. The upper border of the symphysis pubis is the most correct and unchangeable fixed point from which to measure the height of the fundus uteri, beginning at that point of pregnancy when the womb can be distinctly felt above the pubes.

3. The height of the fundus uteri above the pubes is a trustworthy objective symptom of the various periods of pregnancy in normal and in reducible oblique presentation, when the womb contains only one fœtus. In these cases it is necessary to note the presenting part of the fœtus and the size of the pelvis.

4. For plural pregnancies a different scale of measurement should be adopted.

5. In non-reducible oblique, and transverse presentations, the height of the womb cannot serve to indicate the period of pregnancy. In these cases the length of the fœtus, determined according to Ahfield's method, will serve to point out the corresponding height of the fundus uteri.

6. In slightly narrowed pelves the distance between the fundus uteri and symphysis pubis is almost equal to the height in breech presentations. In cases of greater pelvic contraction we have nothing to rely upon but the state of development of the fœtus as determined by Ahfield's method.

III. Dr. Rheinstaedter summarizes his views as to the diagnosis of ovarian cysts as follows:

1. The presence of paralbumen does not at all prove the existence of hydrovarium.

2. From its absence we cannot argue with certainty the non-existence of cystic disease of the ovary.

3. There is great probability of a hydrovarium if we find paralbumen abundant, with a viscid condition of the fluid, like barley water, and with an abundant deposit of cellular detritus, and large round cells that are swollen or undergoing fatty degeneration.

4. The presence of well-formed, nucleated cells of cylindrical epithelium collected in groups or rows, speaks positively for hydrovarium, especially when this microscopic investigation agrees with the gross appearances and chemical constitution of the fluid.

In reference to treatment he says: A woman with a moderately large ovarian cyst which is stationary, should not be operated upon unless she urgently desires it. An expectant treatment should be adopted. Extirpation is indicated in rapidly growing cysts, also in suppuration of the cyst. He regards punctures of small cysts, firmly attached within the pelvis, through the vaginal walls, with drainage, favorably. Ovariotomy is sometimes indicated in pregnancy, and should be adopted in preference to abortion or premature delivery.

